# EGFR as a stable marker of prostate cancer dissemination to bones

**DOI:** 10.1038/s41416-020-01052-8

**Published:** 2020-09-09

**Authors:** Paulina Nastały, Sara Stoupiec, Marta Popęda, Julia Smentoch, Thorsten Schlomm, Colm Morrissey, Anna Joanna Żaczek, Burkhard Beyer, Pierre Tennstedt, Markus Graefen, Elke Eltze, Paolo Maiuri, Axel Semjonow, Klaus Pantel, Burkhard Brandt, Natalia Bednarz-Knoll

**Affiliations:** 1 Laboratory of Translational Oncology, Institute of Medical Biotechnology and Experimental Oncology, Medical University of Gdańsk, Gdańsk, Poland; 2 FIRC (Italian Foundation for Cancer Research) Institute of Molecular Oncology (IFOM), Milan, Italy; 3 Department of Tumour Biology, University Medical Centre Hamburg-Eppendorf, Hamburg, Germany; 4 Department of Urology, Charité University Hospital Berlin, Berlin, Germany; 5 Department of Urology, University of Washington, Seattle, WA USA; 6 Martini-Clinic, Prostate Cancer Center and Section for Translational Prostate Cancer Research at the Clinic of Urology at University Medical Center Hamburg-Eppendorf, Hamburg, Germany; 7 Institute of Pathology Saarbruecken-Rastpfuhl, Saarbruecken, Germany; 8 Department of Urology, Prostate Center University Clinic Münster, Münster, Germany; 9 University Medical Centre Schleswig-Holstein, Kiel, Germany

**Keywords:** Metastasis, Tumour biomarkers

## Abstract

**Background:**

Prostate cancer (PCa) is among the most commonly diagnosed malignancies in men. Although 5-year survival in patients with localised disease reaches nearly 100%, metastatic disease still remains incurable. Therefore, there is a need for markers indicating metastatic dissemination.

**Methods:**

EGFR overexpression (EGFR^over^) was tracked in 1039 primary tumours, circulating tumour cells from 39 d’Amico high-risk patients and metastatic samples from 21 castration-resistant PCa cases. EGFR status was compared to clinical parameters and multiple molecular factors were assessed using immunohistochemistry and gene ontology analysis. The functional aspect of EGFR was evaluated by plating PC-3 cells on soft and rigid matrices.

**Results:**

EGFR^over^ was found in 14% of primary tumours, where it was associated with shorter metastasis-free survival and was an independent indicator of worse overall survival. EGFR^over^ correlated with a pro-migratory and pro-metastatic phenotype of tumour cells as well as rich collagen fibre content. All circulating tumour cells (detected in 13% of cases) were positive for EGFR, independent of their EMT-related phenotype. EGFR^over^ was more prevalent in castration-resistant bone metastases (29% of patients) and supported growth of human PCa cells on rigid matrices mimicking bone stiffness.

**Conclusions:**

EGFR^over^ is a stable, EMT-independent marker of PCa disseminating to rigid organs, preferentially bones.

## Introduction

Prostate cancer (PCa) is the second most frequent malignancy in men worldwide.^1^ Although 5-year survival in patients with localised PCa is nearly 100%, metastatic disease still remains incurable.^2,3^ Therefore, there is an urgent need for markers that could help to detect initial stages of tumour dissemination, probability of recurrence and predict preferred sites of metastasis in order to personalise patients’ treatment.

Epithelial–mesenchymal transition (EMT) and plasticity are involved in metastatic progression of PCa.^4–6^ In addition, significant roles for the epidermal growth factor receptor (EGFR) have been suggested in prostate tumorigenesis and progression.^7,8^ EGFR expression was previously shown to be associated with high grade, advanced stage and high risk for prostate-specific antigen (PSA) recurrence^9^ and bone metastases.^10^ In addition, EGFR was also shown to control bone development.^11^ Indeed, as one of the regulators of EMT, (de)differentiation, proliferation and angiogenesis, EGFR might initiate and/or promote tumour dissemination and metastasis and thus may be considered as a surrogate marker of high metastatic potential.^10^ However, there is a lack of a complex study evaluating EGFR expression in PCa in the context of tumour characteristics and at various stages of PCa dissemination.

Thus, in the current study, the expression of EGFR protein was assessed in the dissemination cascade—throughout the disease process from primary tumours to disseminated circulating tumour cells (CTCs) and metastatic samples obtained from castrate-resistant PCa (CRPC) patients at the time of death. It was also compared to clinical parameters and multiple molecular factors (including EMT-related proteins, collagen fibre content, vascular and lymphatic vessels numbers) to evaluate its feasibility as a stable marker in the PCa dissemination process.

## Methods

### PCa patients of cohort I to study primary tumours

One-thousand two hundred PCa patients were included in this study (Supplementary Table 1) based on their signed informed consent, after the approval of the local Ethics Committee (i.e. Ethik Kommission der Aerztekammer Westfalen-Lippe und der Medizinischen Fakultaet der Westfaelischen Wilhelms-Universitaet Muenster, Germany, no. 2007–467–f–S). The patients underwent radical prostatectomy at the Department of Urology in the Prostate Centre University Clinic Münster (Germany) between 1993 and 2004. The variable clinico-pathological and molecular parameters were documented as described.^12,13^ Time to biochemical recurrence was defined as time between prostatectomy and the time point of first serum PSA increase >0.1 ng/mL followed by another value >0.1 ng/mL after surgery. Metastasis-free survival was defined as the time between prostatectomy and occurrence of clinically defined metastases. Overall survival was defined as the time between the prostatectomy and patient death. Last follow-up was completed in June 2019. The median follow-up was 76 months (range 0.1–273 months).

### PCa patients of cohort II to study CTCs

Fifty-nine d’Amico high-risk PCa patients treated in the Martini-Clinic at the University Medical Center Hamburg-Eppendorf (Hamburg, Germany) between 2012 and 2013 were enrolled in this study after informed consent based on the approval of the local ethical review board number PV3779 as presented in Supplementary Table 2. Blood samples (at mean volume of 7.5 mL, range 5–12 mL; first 2 mL of collected blood discarded to avoid contamination by skin cells) were collected into EDTA tubes. Last follow-up was completed in September 2015. The median observation time was 13 months (range 1–25 months).

### PCa patients of cohort III to study metastases

Visceral and bone metastases were obtained from 21 PCa patients who died of metastatic CRPC and who signed written informed consent for a rapid autopsy performed within 6 h of death, under the aegis of the Prostate Cancer Donor Program at the University of Washington and approved by the Institutional Review Board of the University of Washington (Supplementary Table 3).

### Tissue microarrays (TMAs)

TMAs with primary or metastatic PCa samples were prepared as previously described.^13,14^

### The Cancer Genome Atlas (TCGA) prostate adenocarcinoma (PRAD) data set

RNA-seq data (RNASeqV2, RSEM normalised) covering normalised counts of sequences aligning to 20,531 genes were obtained for 497 PRAD patients from TCGA portal (data status of 28 January 2016). The methods of biospecimen procurement, RNA isolation and RNA sequencing were previously described by TCGA Research Network.^15^

### Isolation of CTCs

Peripheral blood was processed within 24 h of collection. Peripheral blood mononuclear cell (PBMC) fraction, preferably containing CTCs, was enriched using Ficoll density gradient centrifugation, resuspended in 5 mL of 1× phosphate-buffered saline (PBS) and centrifuged to prepare microscopic slides, each containing 500,000 cells. The slides were left overnight to air-dry at room temperature and used within 24 h for further CTC analysis.

### Immunohistochemical (IHC) detection and evaluation of EGFR

To detect EGFR, TMA sections were deparaffinised and treated with Proteinase K Ready-to-Use (Dako) for 6 min and Perioxidase-Blocking Solution (Dako) for 5 min. TMAs were incubated overnight at 4 °C with mouse monoclonal anti-EGFR in vitro diagnostic antibody (E30, Dako) diluted 1:20, envisioned by EnVision Kit, Rabbit/Mouse (Dako) and counterstained with haematoxylin (Merck, Germany). The intensity (negative, weak, moderate or strong), subcellular localisation of the staining (membranous, cytoplasmic, nuclear) and the percentage of positive tumour cells were documented. Two tumour samples (TMA tissue cores) from each patient were assessed individually by two independent observers, experienced in IHC analysis. The EGFR intensity (negative, weak, moderate, strong) was evaluated according to the analogical recommendations for HER2 testing in breast cancer proposed by American Society of Clinical Oncology.^16^ To evaluate overall score corresponding to one patient, maximal intensity of EGFR was chosen from two analysed tumour samples. If one tissue core was uninformative, the overall score corresponded to the remaining one.

### IHC detection and evaluation of other proteins

IHC for vimentin, epithelial cell adhesion molecule (EpCAM) and keratins K8/18 and K19 was performed, evaluated and categorised as negative vs. positive staining as described.^13,14^ The number of vascular or lymphatic vessels was examined in each tumour sample as the number of vessels with visible lumen, positive for CD34 and podoplanin staining, respectively.^12^

### EGFR/EpCAM/pan-keratin/CD45 immunocytochemical staining on CTCs

Immunocytochemical staining identifying CTCs was performed for each patient on 3 slides containing 500,000 PBMCs each. The slides were fixed for 10 min with the Fixation Solution B for Epithelial Cell Detection Kit (Micromet AG; 135 μL diluted in 10 mL of 1× PBS), incubated for 5 min with Peroxidase-Blocking Solution (Dako) and subsequently for 20 min with AB blocking serum (Bio-Rad Medical Diagnostics) diluted 1:10 in 1× PBS. CD45 was detected by incubation with a mouse antibody (NCL-LCA-RP, Novocastra, diluted 1:100, 45 min) and secondary rabbit polyclonal anti-mouse antibody labelled with horseradish peroxidase (Dako, diluted 1:100, 30 min) followed by addition of DAB substrate (Dako, diluted 1:50, 10 min). EGFR was detected by incubation with rabbit polyclonal antibody (sc-03, SantaCruz, diluted 1:100, overnight at +4 °C) and secondary anti-rabbit antibody labelled with Alexa 555 (Life Technologies, diluted 1:200, 45 min). EpCAM was detected by incubation with mouse antibody (NCL-ESA, Novocastra, diluted 1:100, 45 min) and secondary anti-mouse Alexa 350–conjugated antibody (Life Technologies, diluted 1:200, 45 min). Subsequently, cells were incubated for 45 min with anti-pan-keratin antibody AE1/AE3 (eBioscience, diluted 1:700) and C11 (Cell Signalling Technology, diluted 1:300) both directly labelled with Alexa 488. Nuclei of the cells were counterstained with Red-Dot (Biotum, diluted 1:200, 30 min) and covered with coverslips with one drop of Moviol (Sigma Aldrich). Three slides per patient were screened and evaluated under the fluorescence microscope (Axioplan2) in five fluorescent channels and brightfield for putative CTCs under magnification ×400 and ×600. A cell was classified as a CTC based on its cellular and nuclear morphology (inclusion criteria: intact and non-apoptotic cell morphology, intact non-leucocyte-like nucleus, non-granulocyte-like morphology, cell diameter of minimum 5 µm) and absence of CD45 staining. Keratins, EGFR and EpCAM expression was evaluated in such cells and documented. If no CTCs were found, a subsequent three slides were stained and analysed to confirm CTC status in such patients.

### Collagen fibre content evaluation

Collagen fibres were visualised with polarised light microscopy^17^ using Olympus BX63 microscope equipped with a camera (Leica DFC450C) and ×10 objective (UPlanSApo, NA 0.4). The percentage content of collagen fibres was evaluated and divided into 3 groups—low (<25%), moderate (25–50%) and high (>50%)—and correlated to other molecular markers.

### Gene ontology analysis

The patients from TCGA PRAD data set were categorised into four subgroups according to the normalised mRNA expression of *EGFR* and α-1 type I collagen (*COL1A1*). For both genes, patients were dichotomised according to the third quartile (Q3) cut-off. Further, phenotypes *EGFR*^positive^*COL1A1*^positive^ (*n* = 31, 6%), *EGFR*^negative^*COL1A1*^positive^ (*n* = 93, 19%), *EGFR*^positive^*COL1A1*^negative^ (*n* = 93, 19%) and *EGFR*^negative^*COL1A1*^negative^ (*n* = 280, 56%) were defined in those patients. Differences in gene expression between the 4 subgroups were estimated with Kruskal–Wallis test with Benjamini–Hochberg correction; *p* values < 0.05 and false discovery rate values < 0.05 were considered statistically significant. Low-expression genes (median count in each group = 0 and median count in all samples < 100) were excluded, leaving 11,075 transcripts for further analysis. For the *EGFR*^positive^*COL1A1*^positive^ group, transcripts with the lowest/highest expression in comparison to the three remaining groups were selected based on expression medians. Selected genes were associated with functional annotations using the Functional Annotation Tool by DAVID Bioinformatics Resources 6.8.^18,19^ EASE Score, a modified Fisher’s exact *p* value, was used to assess gene enrichment. Multiple testing was corrected using Benjamini correction. The data were analysed using the R statistical environment (3.6.1).^20^

### PCa cell culture and fluorescence-activated cell sorting (FACS) analysis

PCa cell line PC-3 was obtained from ATCC (American Type Culture Collection) and cultured in Ham’s F12 (Biowest), supplemented with 10% foetal bovine serum (Euroclone) and 2 mM L-Glutamine (Euroclone). For FACS analysis aiming to separate EGFR^high^ and EGFR^low^ subpopulations, the cells were trypsinised with 0.05% trypsin (Life Technologies) for 5–10 min and incubated with 5 µg/mL anti-EGFR antibody (Thermo Fisher, clone 30H45L48) for 30 min on ice. After washing in 1×PBS, the cells were incubated for 30 min on ice with secondary anti-rabbit antibody conjugated with Alexa-488 (Jackson Immuno Research, Cat. 211-542-171). Then the samples were sorted using FACSAria using 488 nm laser (BD Bioscience) and analysed with the BD FACSDiva 8.0.1 software (BD Bioscience, IFOM license).

### Cell plating and their outgrowth measurement on matrices with different rigidity

Two distinct populations of high or low EGFR PC-3 cells sorted by FACS were plated on hydrogels characterised by different rigidity (i.e. 0.2, 2, 8 and 25 kPa) bound to 6-well glass bottom plates (Cell Guidance Systems), previously coated with 0.01 mg/mL rat tail collagen type I (Corning), and incubated for 48 h under standard culture conditions followed by fixation for 10 min in 4% paraformaldehyde/1×PBS. The cells were permeabilised in 0.1%Triton-X/1×PBS, incubated in blocking solution (1% bovine serum albumin in 1×PBS) and primary anti-Ki-67 antibody (Abcam, ab16667). Afterwards, a secondary anti-rabbit antibody conjugated with Alexa-488 (Jackson ImmunoResearch) was added for 45 min. Nuclei were stained with NucBlue dye (Hoechst 33342, Thermo Fisher). The samples were kept in 1×PBS at 4 °C until microscopic analysis. The images were acquired with Olympus 1×81 microscope, equipped with ×10 objective and cellSens software. At least 50 cells from 7 different fields of view were imaged. The single nuclei and Ki-67-positive cells were counted. The cells from clusters were analysed only when the nuclei were separable. The percentage of Ki-67-positive cells was evaluated and analysed using the Prism software.

### Statistics

Statistical analysis was performed using SPSS version 25.0 licensed for the University of Gdańsk. Chi-square or Fisher’s exact tests were used in order to compare EGFR expression to molecular factors and clinico-pathological parameters. Mann–Whitney test was used to compare continuous variables. Associations between EGFR expression and time-to-biochemical recurrence or time-to-death were evaluated using log-rank test and presented as Kaplan–Meier plots. To estimate hazard risk, Cox hazard potential regression analysis (95% confidence interval (CI)) was performed. Cases with missing values were excluded from the study or subanalyses. Uninformative or technically damaged samples were excluded from the analyses. All results were considered statistically significant if *p* < 0.05. The study was conducted according to REMARK study recommendations,^21^ in accordance with the Helsinki Declaration of 1975 and STROBE checklist.^22^

## Results

### EGFR overexpression is associated with poor prognosis in PCa patients

**Fig. 1:**
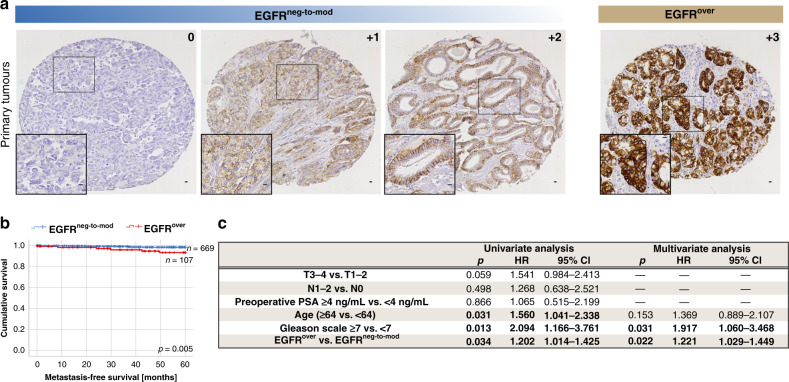
Clinical significance of EGFR overexpression in primary tumours. **a** Representative immunohistochemical staining of EGFR in primary prostate tumours, scale bar 100 μm. **b** Kaplan–Meier estimates of metastasis-free survival, *n* = 776. **c** Univariate and multivariate analysis of overall survival.

Membranous and membranous/cytoplasmic expression of EGFR with different intensity was evaluated in 1841 primary PCa samples from 1033 patients. EGFR staining intensity was divided into two groups—negative/weak/moderate (EGFR^neg-to-mod^) and strong (EGFR^over^; Fig. 1a)—present in 890 (86.2%) and 143 (13.8%) patients, respectively. Interestingly, in primary tumours EGFR^over^ was not associated with EGFR gene dosage (and specifically EGFR gains) assessed by fluorescent in situ hybridisation (*n* = 277, Supplementary Fig. 1a). It also did not correlate to the shorter (≤18) length of CA repeats in intron I of EGFR gene^23^ in the small subset of primary prostate carcinomas (*n* = 26, data not shown). In addition, there was no correlation between EGFR expression and clinico-pathological parameters (i.e. age, T status, N status, preoperative PSA and Gleason score) in the whole cohort of patients (data not shown).

At landmark time points of 5 months, among patients with EGFR^over^, metastasis-free survival was significantly shorter than in the EGFR^neg-to-mod^ group [(*n* = 776, Kaplan–Meier log-rank analysis, *p* = 0.005), Fig. 1b]. This result was even more pronounced in patients who did not reach a PSA concentration <0.1 ng/mL [(*n* = 59, Kaplan–Meier log-rank analysis, *p* = 0.018), Supplementary Fig. 1b] or d’Amico high-risk group [(*n* = 517, Kaplan–Meier log-rank analysis, *p* = 0.001), Supplementary Fig. 1c]. In addition, EGFR^over^ was a marker of poor overall survival in both univariate (Cox, *p* = 0.034, hazard ratio (HR) = 1.202, 95% CI = 1.014–1425, Fig. 1c) and multivariate analysis (Cox, *p* = 0.022, HR = 1.221, 95% CI = 1.029–1.449, Fig. 1c).

### EGFR overexpression correlates with pro-migratory and pro-metastatic phenotype of tumour cells

**Fig. 2:**
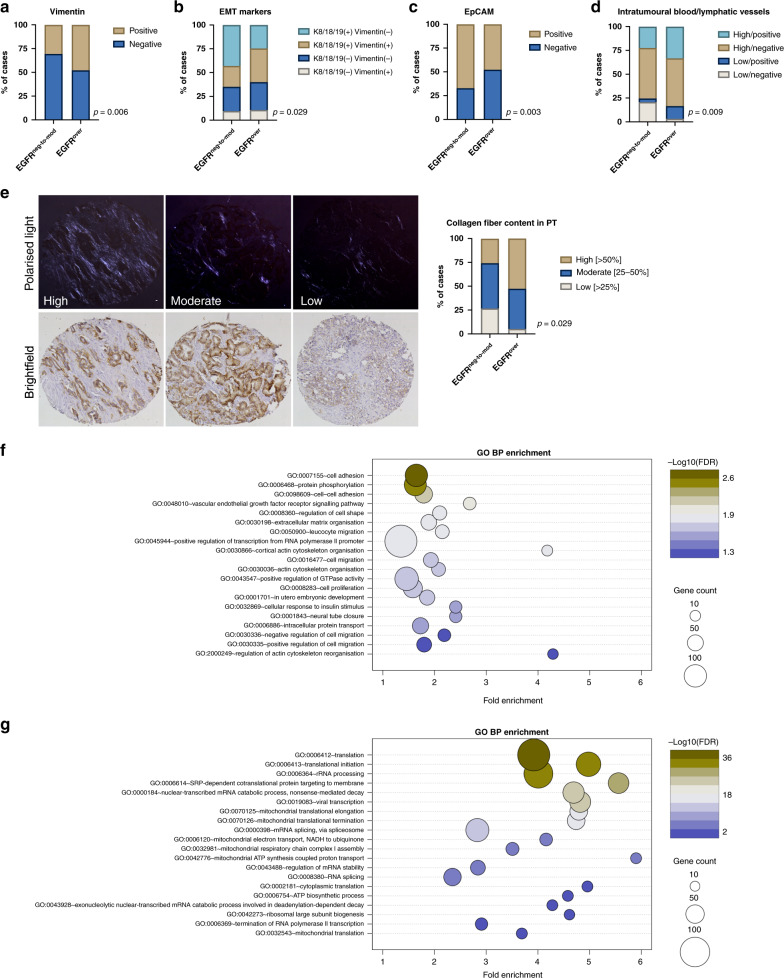
Characteristics of EGFR overexpression in primary tumours. **a** Vimentin expression in EGFR^neg-to-mod^ and EGFR^over^ cases, *n* = 415 tumours. **b** Expression of EMT-related markers in EGFR^neg-to-mod^ and EGFR^over^ cases, *n* = 383 tumours. **c** EpCAM expression in EGFR^neg-to-mod^ and EGFR^over^ cases, *n* = 501 tumours. **d** Prevalence of blood and lymphatic vessels in EGFR^neg-to-mod^ and EGFR^over^ cases, *n* = 472 tumours. **e** Representative images of collagen content quantification (left panel), collagen content distribution in EGFR^neg-to-mod^ and EGFR^over^ cases, *n* = 120 patients. **f** GO BP terms enriched in genes upregulated in *EGFR*^positive^*COL1A1*^positive^ tumours; top 20 terms with the lowest *p* value plotted against fold enrichment and ordered according to −log10(FDR); dot size represents the number of genes associated with the term, dot colour represents −log10(FDR); analysed with Functional Annotation Tool by DAVID Bioinformatics Resources 6.81. **g** GO BP terms enriched in genes downregulated in *EGFR*^positive^*COL1A1*^positive^ tumours; top 20 terms with the lowest *p* value plotted against fold enrichment and ordered according to −log10(FDR); dot size represents the number of genes associated with the term, dot colour represents −log10(FDR); analysed with Functional Annotation Tool by DAVID Bioinformatics Resources 6.81.

EGFR^over^ correlated with the expression of mesenchymal cell marker vimentin (*n* = 415, Chi^2^ = 7.632, *p* = 0.006, Fig. 2a), intermediate epithelial–mesenchymal phenotype^24^ characterised by phenotype K8/18/19(+)vim(+) and K8/18/19(−)vim(−), (*n* = 393, Chi^2^ = 9.002, *p* = 0.029, Fig. 2b) and the loss of expression of EpCAM (*n* = 501, Chi^2^ = 8.645, *p* = 0.003, Fig. 2c). Moreover, EGFR^over^ occurred more frequently in the tumours with a higher number of intratumoural lymphatic vessels and blood vessels assessed using podoplanin and CD34 staining, respectively (*n* = 472, Chi^2^ = 11.541, *p* = 0.009, Fig. 2d and Supplementary Fig. 1d, e).

In primary tumours from 120 PCa patients, percentage content of the collagen fibre content was quantified. A high collagen fibre content (>50%) was associated with the EGFR^over^ phenotype of tumour cells (*n* = 120, Chi^2^ = 7.114, *p* = 0.029, Fig. 2e).

With the gene ontology analysis, multiple genes involved in cell migration, adhesion and proliferation, as well as angiogenesis regulation, were significantly upregulated in tumours expressing *EGFR* and *COL1A1* (Fig. 2f and Supplementary Data 1). On the contrary, multiple genes involved in translation, transcription and mitochondrial metabolism were downregulated in this subgroup of tumours (Fig. 2g and Supplementary Data 2).

### EGFR is an EMT-independent marker of CTCs in d’Amico high-risk PCa patients

**Fig. 3:**
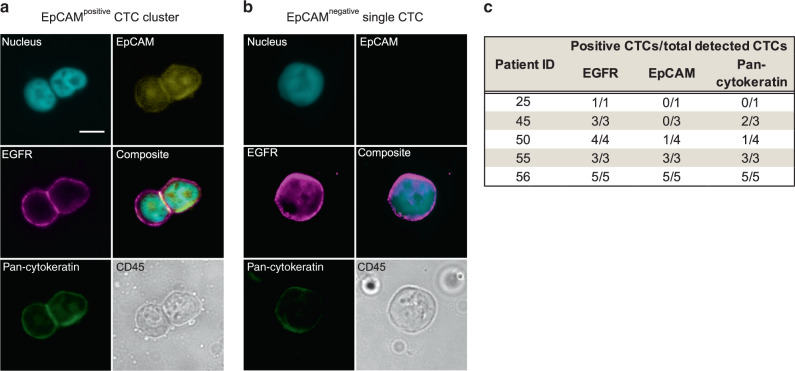
EGFR overexpression in circulating tumour cells from d’Amico high-risk patients. **a** Representative CTC cluster composed of two cells positive for EGFR, pan-keratin and EpCAM and negative for CD45; scale bar 10 μm. **b** Representative single CTC positive for EGFR and pan-keratin and negative for EpCAM and CD45. **c** Characteristics of CTCs detected in d’Amico high-risk patients. Number of CTCs expressing EGFR, EpCAM and pan-keratin/total detected CTCs.

Single CTCs (*n* = 11) and CTC clusters (*n* = 2) were isolated from 5 (12.8%) of 39 analysed d’Amico high-risk patients. The CTC yield varied between 1 and 5 CTCs/1,500,000 PBMCs per patient. All detected CTCs showed strong EGFR and were negative for CD45 (Fig. 3a–c and Supplementary Fig. 2a). Other common CTC markers including pan-keratins and EpCAM were also evaluated. Only three patients had CTCs positive for EpCAM, including all cells from both clusters (Fig. 3a, c). Pan-keratin status of the detected CTCs was heterogonous, varying from negative (five cells) through weak (six cells) to moderate expression—five cells in two clusters (Fig. 3a–c). CTCs’ yield and phenotype were not associated with any tumour characteristics (Supplementary Table 4). Despite the short observation time after surgery (≤25 months) and limited follow-up cohort, patients with EGFR^over^ CTCs had significantly shorter time to biochemical recurrence and time to metastasis than patients negative for CTCs (*n* = 25, Kaplan–Meier log-rank analysis, *p* = 0.002 and *p* < 0.001, respectively, Supplementary Fig. 2b, c). In addition, 3 of those 5 patients with EGFR^over^ CTCs developed distant metastases to lymph nodes (patient with CTCs negative for EpCAM) or bones (patient with weakly positive CTCs for EpCAM), whereas none of the patients developed metastasis in the cohort negative for CTCs (*n* = 23, Fisher’s exact test, *p* = 0.005).

### EGFR overexpression is most frequent in CRPC metastases to bones

**Fig. 4:**
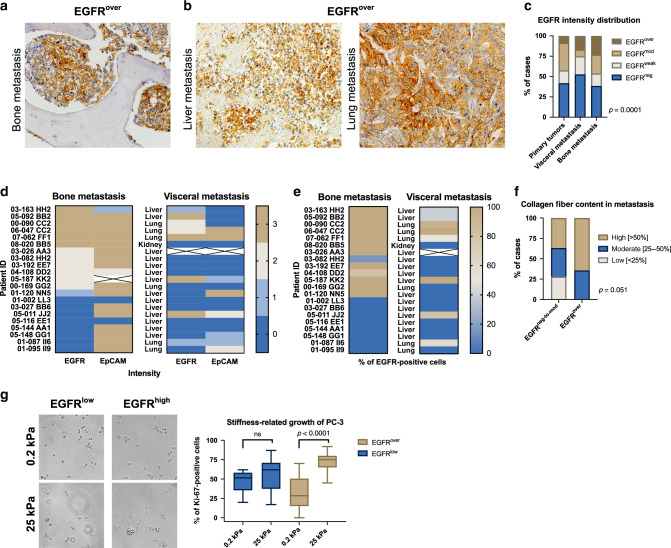
Characteristics of EGFR overexpression in metastases. **a** Representative immunohistochemical staining of EGFR^over^ in bone metastasis. **b** Representative immunohistochemical staining EGFR^over^ in visceral metastases. **c** EGFR staining intensity distribution in primary tumours (*n* = 1841), visceral metastases (*n* = 36) and bone metastases (*n* = 39). **d** EGFR and EpCAM staining intensity distribution in bone and visceral metastases. **e** Percentage of EGFR-positive cells in bone and visceral metastases. **f** Collagen content distribution in EGFR^neg-to-mod^ and EGFR^over^ metastases (*n* = 71). **g** Representative images of EGFR^low^ and EGFR^high^ PC-3 cell growth on soft (0.2 kPa) and rigid (25 kPa) matrices. Quantification of PC-3 cells proliferation using Ki-67 marker on soft (0.2 kPa, *n* = 70 cells) and rigid (25 kPa, *n* = 91 cells) matrices.

Membranous and membranous/cytoplasmic EGFR expression was also evaluated in 75 tissue cores from castration-resistant metastases of 21 patients (Fig. 4a, b). EGFR expression was significantly more frequent in castration-resistant bone metastases, when compared to its distribution in primary tumours (*n* = 39 vs. *n* = 1841, Chi^2^ = 11.543, *p* = 0.009, Fig. 4c, d). Interestingly, also EpCAM strong intensity reached 76% in bone metastases from CRPC patients (Fig. 4d). In bone metastases, the percentage of EGFR-positive tumour cells frequently reached 100% per tumour sample (Fig. 4e) and its mean value was significantly higher than in the cohort of EGFR-positive primary tumours (92%, *n* = 13 vs. 58%, *n* = 1258; two-tailed Mann–Whitney test, *p* < 0.0001). There was no correlation between EGFR^over^ and EpCAM, K8/18/19 and vimentin expression in castration-resistant bone and/or visceral metastases (data not shown). In addition, there was a borderline correlation between higher prevalence of collagen fibres (>50%) and the EGFR^over^ phenotype of tumour cells in metastases (*n* = 71, Chi^2^ = 5.934, *p* = 0.051, Fig. 4f).

### High expression of EGFR improves PC-3 proliferation on rigid matrices

On collagen-coated rigid matrices (25 kPa), mimicking bone tissues rigidity, PC-3 cells sorted according to their high EGFR expression, adhered and grew more efficiently than sub-lines with low EGFR expression (two-tailed Mann–Whitney test, *p* < 0.0001, Fig. 4g and Supplementary Fig. 3a, b). On the other hand, such increase in adhesion and growth was not observed on soft substrate (0.2 kPa). Interestingly, PC-3 cells plated on matrices with different rigidity (0.2 and 25 kPa) revealed no significant change in the epithelial/mesenchymal phenotype characterised by K19/vimentin ratio assessed by real-time PCR (data not shown).

## Discussion

Our data indicate that EGFR^over^ might be a candidate biomarker of stable marker of PCa dissemination cascade.

EGFR overexpression, found both in primary tumour and CTCs, was an indicator of poor prognosis. In primary tumours, it was associated with shorter metastasis-free survival, which has been previously shown to be related with a significant risk of death from PCa.^25^ Our observation is similar to the ones from other groups,^9^ performed, however, mainly in CRPC.^26,27^

The expression of EGFR was reported to be associated with EMT,^28,29^ which promotes pro-migratory and pro-survival behaviour of tumour cells, generating their aggressive phenotype.^4^ Based on our data, EGFR overexpression correlated with pro-migratory and pro-metastatic phenotype of PCa tumour cells. In EGFR^over^ tumours, we also found a higher collagen fibre content that can influence cell migration, invasiveness and proliferation and indicate worse prognosis,^30,31^ Gene ontology analysis further confirmed that cancers with high expression of both *EGFR* and gene-encoding type I collagen have upregulated genes involved in cell migration and adhesion. Tumours with strong EGFR expression had a larger number of blood and/or lymphatic vessels that could facilitate haematogenous and lymphatic dissemination of cancer cells.^32^

Of note, EMT is a phenomenon hindering detection of CTCs in bloodstream.^33–35^ Our results suggest that EGFR seems to be a stable signature of PCa progression, which might serve as surrogate marker of CTCs undergoing EMT. Importantly, based on our collected data, in a d’Amico high-risk patient cohort, inclusion of EGFR can improve both CTC detection and stratification of patients. However, this result needs further confirmation in a larger cohort of patients as the absolute number of CTC-positive patients and actual number of CTCs in the present study are too low to allow for strong conclusions.

In our study, EGFR^over^ was enriched in bone metastases, suggesting that organ-specific factors such as its stiffness or tumour microenvironment might result in regulation of harbouring and/or nesting of EGFR-positive tumour cells. PCa cells that disseminate show an exquisite tropism for the bone.^36^ In an autopsy study, 90% of the men who had died with haematogenous metastases of PCa were diagnosed with bone metastases.^37^ However, the possible molecular mechanisms involved in governing bone metastases tropism is still poorly understood.^36^ Bone is among tissues characterised by elastic moduli with the greatest stiffness.^38^ It was also reported that EGFR can be involved in rigidity sensing after associating with nascent adhesions under rigidity-dependent tension.^39^ Moreover, human squamous cell carcinoma cells, in response to matrix stiffening, increased EGFR expression and invasiveness.^40^ In our study, EGFR positively correlated with collagen fibre content that can indicate tumour stiffness.^40,41^ Together with our data showing improved proliferation of EGFR-overexpressing PCa cells on rigid matrices, it can be speculated that EGFR can promote growth of cells on rigid substratum and bone metastases.

To sum up, the data collected within this study suggest that EGFR is a marker of PCa dissemination, independent of EMT.

## Supplementary information

Supplementary Information

Supplementary Dataset 1

Supplementary Dataset 2

## Data Availability

The data that support the findings of this work are available from the corresponding author upon request.
