# Supplementary material for: EGFR as a stable marker of prostate cancer dissemination to bones
Source: Br J Cancer. 2020 Sep 9;123(12):1767–74. doi: 10.1038/s41416-020-01052-8 (PMC7722745; doi:10.1038/s41416-020-01052-8)
Supplement: Supplementary file 1 — Supplementary Information [file 41416_2020_1052_MOESM1_ESM.pdf]

Supplementary Information

EGFR as a stable marker of prostate cancer dissemination to bones

Paulina Nastały, Sara Stoupiec, Marta Popęda, Julia Smentoch, Thorsten Schlomm, Colm Morrissey, Anna J. Żaczek, Burkhard Beyer, Pierre Tennstedt, Markus Graefen, Elke Eltze, Paolo Maiuiri, Axel Semjonow, Klaus Pantel, Burkhard Brandt, Natalia Bednarz-Knoll *

*** Corresponding author:**

Natalia Bednarz-Knoll, Laboratory of Translational Oncology, Institute of Medical Biotechnology and Experimental Oncology, Medical University of Gdańsk, Gdańsk, Poland; phone: 0048 58 3491434, fax: 0048 58 3491445, e-mail: nbk@gumed.edu.pl

This file contains:

Supplementary Methods
Supplementary Figure 1
Supplementary Figure 2
Supplementary Figure 3
Supplementary Table 1
Supplementary Table 2
Supplementary Table 3
Supplementary Table 4
References

Supplementary Methods

EGFR gene dosage assessment using fluorescent in situ hybridization

Fluorescent *in situ* hybridization (FISH) probes were prepared from DNA isolated from BAC clone RP5-1091E12 (AC006977) as described (1). FISH protocol using spectrum orange-labelled EGFR probe and spectrum green-labelled centromere 7 (Abbott Molecular) was performed and evaluated as described (2).

CA repeats assessment in CA-SSRI

DNA was isolated from FFPE specimens (3x sections of 10 μm) as described (1) and CA-SSRI profiling was performed using previously described protocol (3).

RNA isolation and analysis of EMT-related signatures by quantitative real-time PCR

RNA from cancer cell line populations growing on gels with different rigidity was isolated using RNeasy MiniKit (Qiagen, Germany) according to the manufacturer's instructions. Reverse transcription was performed with Anchored-oligo(dT)$_{18}$ Primers using Transcriptor First Strand Synthesis Kit (Roche, Switzerland) and manufacturer's protocol. Gene expression of two reference genes (*ACTB* and *GAPDH*), keratin 19 (*CK19*, Hs01051611_gH) and vimentin (*VIM*, Hs00185584_m1) were examined by quantitative real-time PCR (CFX cycler, Bio-Rad). The commercially available TaqMan assays and Universal PCR Master Mix (Applied Biosystems, USA) were used in qPCR. Briefly, samples (10 ng of cDNA per reaction) were run in triplicates under the following conditions: 2 min at 60°C, 10 min at 95°C, 45 cycles of 1 min at 60°C and 15 sec at 95°C. The data analysis was performed using CFX Manager software (Bio-Rad). The gene expression level was calculated in relation to two reference genes (*ACTB* and *GAPDH*). To validate runs, calibrator (mix of cDNA from MCF-7, MDA-MB-231 cell lines, and PBMCs) was included in each plate to compare the expression levels.

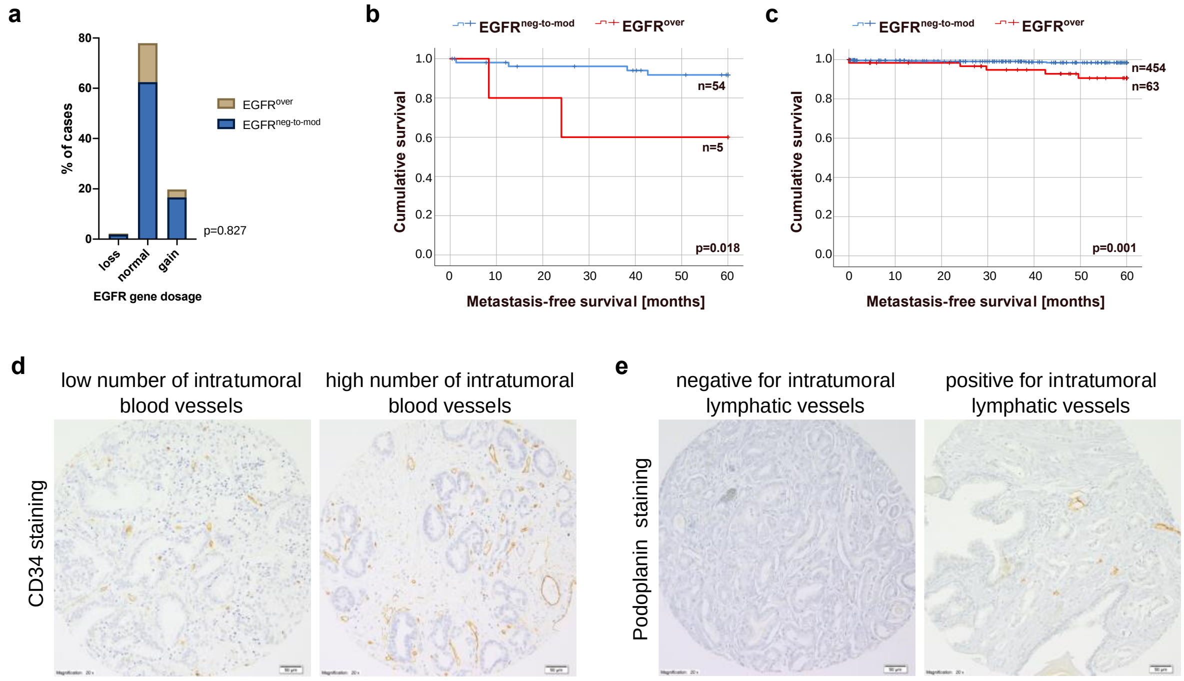

Supplementary Figure 1

Clinical significance of EGFR overexpression in primary tumours

a, EGFR gene dosage in relation to $EGFR_{neg-to-mod}$ and $EGFR_{over}$ intensity. **b**, Kaplan-Meier estimates of metastasis-free survival in patients without PSA decline, n=59. **c**, Kaplan-Meier estimates of metastasis-free survival in d'Amico high-risk patients, n=517. **d**, Representative photomicrographs of CD34 immunohistochemical staining. **e**, Representative photomicrographs of podplanin immunohistochemical staining.

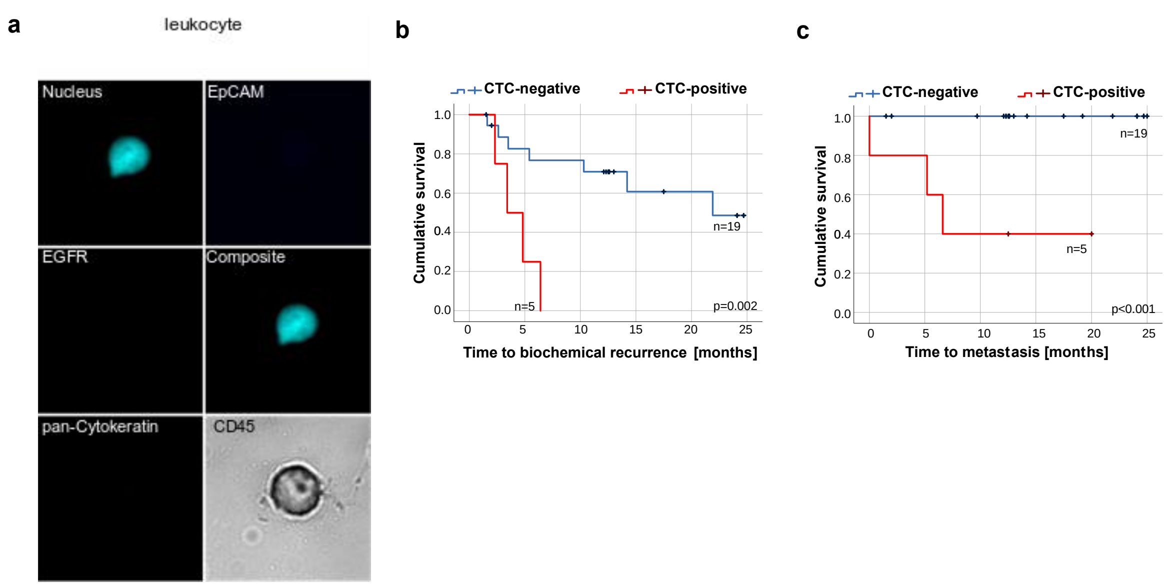

Supplementary Figure 2

EGFR overexpression in circulating tumour cells from d'Amico high-risk patients

a, Representative leukocyte, negative for EGFR, pan-keratin and EpCAM, positive for CD45. **b**, Kaplan-Meier estimates of time to biochemical recurrence for CTC-negative and CTC-positive d'Amico high-risk patients, n=25 patients. **c**, Kaplan-Meier estimates of time to metastasis rates for CTC-negative and CTC-positive d'Amico high-risk patients, n=25 patients.

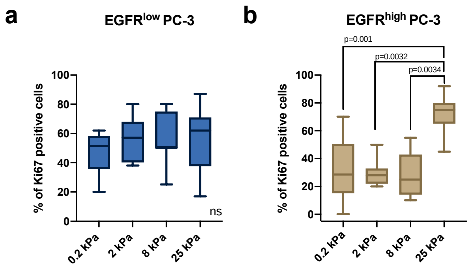

Supplementary Figure 3

Characteristics of EGFR overexpression in metastases.

a, Quantification of $EGFR_{low}$ PC-3 cells proliferation on matrices of different rigidity: 0.2 kPa (n=70 cells), 2 kPa (n=50 cells), 8 kPa (n=50 cells), and 25 kPa (n=91 cells). **b**, Quantification of $EGFR_{low}$ PC-3 cells proliferation on matrices of different rigidity: 0.2 kPa (n=70 cells), 2 kPa (n=50 cells), 8 kPa (n=50 cells), and 25 kPa (n=91 cells).

Supplementary Table 1
PCa patients cohort I to study primary tumour

	Patients	
	n	%
**Age**		
**<64**	624	52.0
**>64**	576	48.0
**total**	1200	
**T status**		
**T2**	501	42.6
**T3**	620	52
**T4**	55	4.7
**total**	1176	
**N status**		
**N0**	1086	92.7
**N1**	80	6.8
**N2**	6	0.5
**total**	1172	
**Gleason Grading Score**		
**<7**	338	28.4
**7**	660	55.5
**>7**	192	16.1
**total**	1190	
**Focality**		
**uni**	152	13.2
**multi**	998	86.7
**total**	1150	
**Capsule penetration**		
**no**	119	10.3
**infiltration**	429	37.2
**penetration**	604	52.4
**total**	1152	
**Margins**		
**negative**	693	60.5
**positive**	453	39.5
**total**	1146	
**Biochemical recurrence**		
**no**	702	72.7
**yes**	264	27.3
**total**	966	
**Metastatic relapse**		
**no**	1146	96.3
**yes**	44	3.7
**total**	1190	
**Death**		
**alive**	1087	91.0
**PCa-related**	18	1.5
**not Pca-related**	53	4.4
**unknown**	36	3.0
**total**	1087	

Supplementary Table 2
PCa patients cohort II to study CTCs

	Patients n	%
**T status**		
**T2a**	2	5.7
**T2b**	12	34.3
**T3a**	13	37.1
**T3b**	8	22.9
**total**	35	
**N status**		
**N0**	24	75.0
**N1**	8	25.0
**total**	32	
**Gleason Grading Score**		
**<7**	5	14.3
**7**	25	71.4
**>7**	5	14.3
**total**	35	
**Biochemical recurrence**		
**no**	13	54.2
**yes**	11	45.8
**total**	24	
**Metastatic relapse**		
**no**	21	87.5
**yes**	3	12.5
**total**	23	

Supplementary Table 3
PCa patients cohort III to study metastases

Patient nr	Gleason score	PSA at diagnosis	Age at Diagnosis	Type of mets		Anti-androgen	Androgen Independence (years)	Androgen ablation duration (years)	Bone met delay from diagnosis (years)	Survival from bone mets (years)	Survival from diagnosis (years)
1	7	42	62	bone	liver	yes	1.1	4.9	11.1	0.4	11.5
2	3+5	11.5	66	bone	lung	yes	3.4	4.0	4.1	0.4	4.5
3	nd	403.5	63	bone	lung	yes	1.0	1.5	0.0	1.7	1.7
4	4+5	245	43	bone	liver	no	2.9	4.0	3.5	0.5	4.0
5	5	6	64	bone	lung	yes	3.8	6.4	3.7	3.8	7.5
6	7	33.9	62	bone	lung	yes	4.9	6.9	4.8	4.8	9.6
7	nd	nd	53	bone	liver	yes	3.1	8.1	5.9	3.5	9.4
8	8	41	63	bone	liver	no	4.4	6.6	5.7	6.9	12.6
9	4+4	54.8	70	bone	liver	yes	1.8	2.8	1.3	1.5	2.8
10	5+4	nd	59	bone	liver	yes	1.4	3.8	2.7	1.2	3.9
11	4+5	2.6	53	bone	liver	yes	bd	5.3	0.0	5.3	5.3
12	3+4	nd	57	bone	liver	yes	1.3	6.8	6.6	0.5	7.1
13	3+4	nd	73	bone	liver	yes	1.2	6.6	9.7	0.8	10.5
14	5+5	nd	75	bone	liver	yes	1.1	6.3	5.6	0.8	6.4
15	4+4	nd	63	bone	liver	yes	7.7	10.6	12.5	5.4	17.9
16	4+5	6	59	bone	liver	yes	0.6	2.2	0.0	2.4	2.4
17	4+5	14	65	bone	liver	yes	1.4	2.2	nd	nd	2.5
18	5+4	105	58	bone	liver	yes	1.5	2.0	0.1	2.0	2.1
19	4+3	12	62	bone	lung	yes	nd	7.3	4.2	3.9	8.1
20	3+3	6.2	70	bone	lung	yes	6.5	9.7	10.5	1.3	11.8
21	3+4	3.3	65	bone	kidney	yes	3.1	8.9	9.0	2.4	11.4

Supplementary Table 4
PCa tumour and patient characteristics in relation to CTC status

	EGFR				
	negative		positive		
	n	%	n	%	Fisher's exact test
**T status**					
**T2a**	2	6.5	0	0	
**T2b**	12	38.7	0	0	
**T3a**	11	35.5	2	50	
**T3b**	6	19.4	2	50	
**total**					0.326
**N status**					
**N0**	22	78.6	2	50.0	
**N1**	6	21.4	2	50.0	
**total**					0.254 (F)
**Gleason**					
**Grading Score**					
**<7**	5	16.1	0	0	
**7**	23	74.2	2	50.0	
**>7**	3	9.7	2	50.0	
**total**					0.083
**Biochemical**					
**recurrence**					
**no**	13	65.0	0	0	
**yes**	7	35.0	4	100	
**total**					**0.031 (F)**
**Metastatic relapse**					
**no**	19	100.0	2	40.0	
**yes**	0	0.0	3	60.0	
**total**					**0.005 (F)**

References

1. Bednarz N, Eltze E, Semjonow A, Rink M, Andreas A, Mulder L, et al. BRCA1 loss preexisting in small subpopulations of prostate cancer is associated with advanced disease and metastatic spread to lymph nodes and peripheral blood. Clin Cancer Res. 2010;
2. Schulte A, Günther HS, Martens T, Zapf S, Riethdorf S, Wülfing C, et al. Glioblastoma stem-like cell lines with either maintenance or loss of high-level EGFR amplification, generated via modulation of ligand concentration. Clin Cancer Res. 2012;
3. Buerger H, Gebhardt F, Schmidt H, Beckmann A, Hutmacher K, Simon R, et al. Length and loss of heterozygosity of an intron 1 polymorphic sequence of egfr is related to cytogenetic alterations and epithelial growth factor receptor expression. Cancer Res. 2000;
